# Prolonged Invasive Mechanical Ventilation is Associated With Decreased Survival After Lung Transplantation Among Recipients With Primary Graft Dysfunction: A Lung Transplant Outcomes Group Study

**DOI:** 10.1111/ctr.70447

**Published:** 2026-01-14

**Authors:** Meghan Aversa, Shaf Keshavjee, Tereza Martinu, Marcelo Cypel, Andrew T. Sage, Joshua M. Diamond, Jonathan P. Singer, Scott M. Palmer, Krishna Pandya, Edward Cantu, Jason D. Christie, Michaela R. Anderson

**Affiliations:** ^1^ University of Toronto Toronto Ontario Canada; ^2^ University of Pennsylvania Philadelphia Pennsylvania USA; ^3^ Glaxo‐Smith Kline London UK; ^4^ University of California San Francisco San Francisco California USA; ^5^ Duke University Durham North Carolina USA; ^6^ University of Kentucky Lexington Kentucky USA

**Keywords:** invasive mechanical ventilation, lung transplantation, primary graft dysfunction

## Abstract

**Introduction:**

Prolonged invasive mechanical ventilation (IMV) after lung transplantation is an appealing early prognostic outcome as it can be reproducibly assessed both prospectively and retrospectively. Whether use of IMV at 72 h after lung transplantation is associated with post‐transplant graft survival is unknown.

**Methods:**

We performed a retrospective cohort study of 1511 participants in the multi‐center Lung Transplant Outcomes Group cohort (2011–2018). Using Cox proportional hazards models and restricted mean survival time, we investigated whether IMV at 72 h was associated with post‐transplant graft survival. We secondarily evaluated whether IMV at 72 h was concordant with severe primary graft dysfunction (PGD).

**Results:**

Participants requiring IMV at 72 h after transplant were sicker at transplantation (higher lung allocation score [LAS], increased extracorporeal membrane oxygenation, or IMV bridge) and more likely to have severe PGD. Use of IMV at 72 h was associated with 55% (95% CI 26%–92%) increased hazards of death or re‐transplantation after adjustment for age, ECMO, diagnosis, LAS, and intra‐operative transfusion. The association between IMV and graft survival was modified by severe PGD (*p*‐for interaction 0.002) but not by pre‐transplant ECMO (*p*‐for interaction 0.88) or pre‐transplant IMV (*p*‐for interaction 0.92). IMV was associated with increased risk of death or re‐transplantation among those with PGD (HR 2.35, 95% CI 1.43–3.85) but not among those without PGD (HR 1.04, 95% CI 0.77‐1.41).

**Conclusion:**

Requirement of IMV at 72 h is an important early post‐transplant outcome associated with post‐transplant survival. This appears driven by those with severe PGD.

**Abbreviations**
BMI: body mass index
BTT: bridge to transplant
CI: confidence interval
CNS: central nervous system
CVA: cerebrovascular accident
ECMO: extracorporeal membrane oxygenation
IMV: invasive mechanical ventilation
IQR: interquartile range
LAS: lung allocation score
PGD: primary graft dysfunction
PH: proportional hazards
PRBC: packed red blood cells

## 1 Background

Prolonged mechanical ventilation after lung transplantation is a potentially useful early outcome for clinical trials in lung transplantation as it can be easily assessed both prospectively and retrospectively with high reproducibility. In one single center study, time to extubation greater than 72 h was associated with increased intensive care unit and hospital length of stay, and increased healthcare costs [1]. In another single center study, time to extubation greater than 14 days was associated with decreased 1‐year survival [2]. Whether time to extubation is associated with overall post‐transplant survival in larger cohorts, across multiple centers, has not been shown.

There are multiple reasons why prolonged mechanical ventilation may be an early marker of poor post‐transplant outcomes. Patients who require invasive mechanical ventilation (IMV) or extracorporeal support as a bridge to transplantation may require prolonged support post‐operatively due to frailty, deconditioning, and multi‐morbidity. Primary graft dysfunction (PGD), allograft injury within 72 h of transplantation, is known to prolong mechanical ventilation due to severe hypoxemia [3, 4, 5, 6, 7]. Patients with post‐operative delirium, stroke, impaired renal or hepatic function, atrial fibrillation, hemorrhage, or other causes of graft dysfunction may also require prolonged intubation.

Using the Lung Transplant Outcomes Group cohort study, we sought to understand whether intubation status at 72 h was associated with post‐transplant survival and to evaluate recipient, donor, and operative characteristics associated with IMV at 72 h. We hypothesized that requiring IMV at 72 h would be associated with decreased post‐transplant graft survival. We secondarily hypothesized that there would be high concordance between use of IMV at 72 h and presence of severe PGD at 48/72 h after lung transplantation.

## 2 Methods

We performed a retrospective cohort study of subjects enrolled in the US‐based multi‐center Lung Transplant Outcomes Group (LTOG) cohort from 2011 to 2018. LTOG prospectively enrolled subjects at nine US lung transplant centers. All participants had prospective clinical data collection pre‐transplant and through the first 72 h after transplant. Vital status was obtained though both manual record review and by report to the United Network for Organ Sharing database. We included subjects with available data on oxygen delivery method at 72 h.

### 2.1 Exposure

Our primary exposure was use of IMV at 72 h after lung transplantation. Use of IMV was obtained prospectively on standardized case report forms and assessed at 0, 24, 48, and 72 h. We used the 72‐h time point due to prior work suggesting median duration of IMV is 48–72 h and prior work investigating 72 h as a viable early endpoint for trials [1, 8, 9].

### 2.2 Outcome

The primary outcome was graft survival defined as time from transplantation to death or re‐transplantation. Subjects who were still alive were censored on October 9, 2021.

### 2.3 Statistical Analysis

We used Cox proportional hazards models to determine the association between IMV at 72 h and allograft survival (death or re‐transplantation). We evaluate the proportional hazards (PH) assumption by regressing Schoenfeld residuals over time. We found that IMV status violated the PH assumption. Therefore, we performed sensitivity analyses to account for this by (1) including of an interaction term between IMV and time and (2) using restricted mean survival time models for 1‐ and 3‐year survival. The interaction term between IMV and time allows us to understand how the association between IMV and survival changes over time. Restricted mean survival time estimates the area under the survival curve over the specified time‐period (i.e., months of graft survival within the specified time‐period for each exposure group) and does not require proportional hazards over time. We report the restricted difference in life expectancy which represents the difference between the survival times for those requiring IMV and those not requiring IMV at 72 h.

We used a directed acyclic graph, developed based on existing knowledge and plausible biologic associations, to identify a minimal set of covariates to address confounding: age, pre‐ or intra‐operative use of extracorporeal membrane oxygenation (ECMO), indication for transplantation, lung allocation score, and intra‐operative packed red blood cell transfusion (Supplemental Figure). A directed acyclic graph allows us to identify minimal set of potential confounders to close all backdoor paths between the exposure and outcome while avoiding inclusion of mediating and colliding variables that might bias causal associations [10]. We stratified Cox models by center to account for differences in the baseline hazards of death by center [11]. Restricted mean survival time models included center as a co‐variate.

We evaluate effect medication by PGD, pre‐transplant ECMO, pre‐transplant mechanical ventilation, and transplant type (single vs. bilateral). Severe PGD was defined as PaO_2_/FiO_2_<200 and radiographic infiltrates at either 48 or 72 h after transplantation (i.e., PGD grade 3 at 48/72 h) [5, 12, 13, 14, 15]. Subjects on ECMO were graded as severe PGD only in the presence of radiographic infiltrates independent of PaO_2_/FiO_2_ ratio [4, 13]. Subjects on ECMO without parenchymal infiltrates were ungradable and excluded from PGD analyses. In sensitivity analyses, we evaluated PGD grade 3 at 72 h [13].

IRB approval was obtained (IRB# 806468). I acknowledge participation in the Transplant Peer Review Network and complied with the journal's author guidelines and policies.

## 3 Results

### 3.1 Participant Characteristics

Of 1528 subjects, 1511 had available data on IMV status at 72 h including 509 (34%) who required IMV at 72 h. Subjects were 59% male with median (Interquartile range) age of 60 (49–66) years, median (IQR) BMI of 25.3 kg/m^2^ (21.8–28.6), 26% had obstructive lung disease, 57% restrictive lung disease, 13% cystic fibrosis, and 4% pulmonary vascular disease. Median (IQR) lung allocation score was 42.0 (35.6–53.8) with 9% requiring ECMO bridge to transplant and 8% requiring IMV bridge to transplant. Seventy‐six percent of subjects underwent bilateral lung transplant.

Subjects who required IMV at 72 h had higher lung allocation score, were more likely to have restrictive lung disease, have required ECMO or mechanical ventilation as a bridge to transplant, and have required intra‐operative extracorporeal life support compared to those who were extubated prior to 72 h (Table 1).

**TABLE 1:** Baseline characteristics by requirement of IMV at 72 h.

	IMV at 72 h (*n* = 509)	No IMV at 72 h (*n* = 1002)
**Recipient characteristics**		
Age (years)	58 (47–65)	61 (51–66)
Male sex	289 (57)	593 (59)
LAS at transplant	47.7 (38.5–74.0)	40.1 (34.6–48.1)
Diagnosis		
Obstructive	89 (18)	302 (30)
Pulmonary vascular disease	40 (8)	27 (3)
Cystic fibrosis	51 (10)	143 (14)
Restrictive	327 (65)	529 (53)
Height (cm)	170.2 (162.5–177.8)	170.2 (162.6–177.8)
Weight (kg)	74.4 (62.6–86.2)	73.0 (60.3–83.9)
BMI (kg/m^2^)	26.2 (22.2–29.5)	25.0 (21.6–28.2)
ECMO BTT	86 (17)	51 (5)
Mechanical ventilation BTT	73 (14)	42 (4)
**Operative characteristics**		
Bilateral transplant	433 (85)	709 (71)
Total ischemic time (min)	640 (492–775)	528 (333–657)
Intra‐op extracorporeal support	243 (48)	181 (18)
Transfusion > 1 L PRBCs	314 (62)	596 (59)
**Donor characteristics**		
Male sex	277 (55)	625 (63)
Donor smoker	216 (48)	390 (44)
Cause of death		
Anoxia	132 (26)	301 (30)
CVA/Stroke	166 (33)	276 (28)
Head trauma	184 (36)	373 (37)
CNS tumor	2 (1)	6 (1)
Unknown/Other	25 (5)	46 (5)
**Post‐transplant outcomes**		
PGD 3 at 48/72 h	268 (55)	112 (11)
PGD 3 at 72 h	202 (47)	59 (6)

Missingness: PGD missing on 33 participants.

Abbreviations: BMI, body mass index; BTT, bridge to transplant; CNS, central nervous system; CVA, cerebrovascular accident; ECMO, extracorporeal membrane oxygenation; LAS, lung allocation score; PGD, primary graft dysfunction; PRBCs, packed red blood cells.

### 3.2 IMV and Survival

**FIGURE 1:**
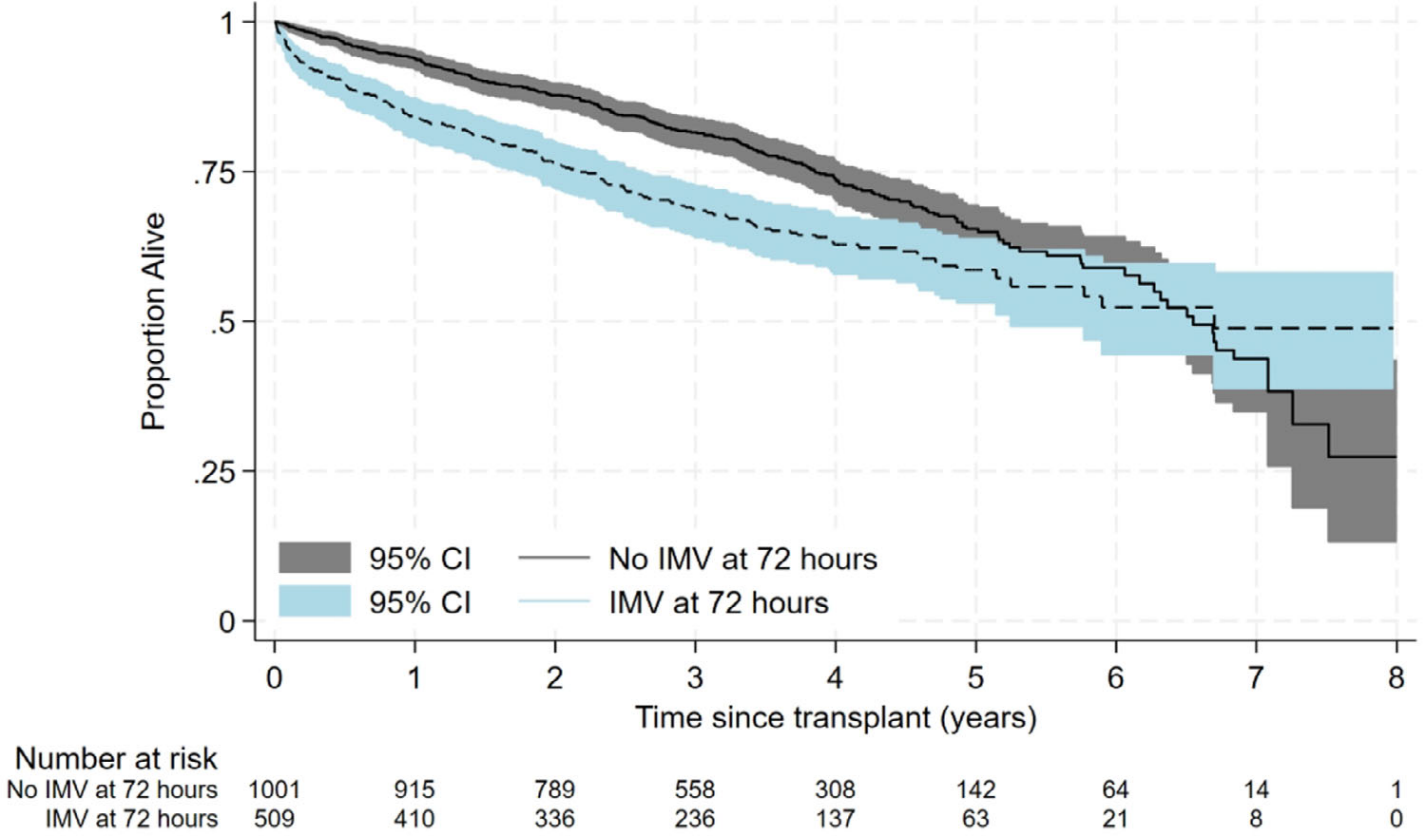
Kaplan–Meier curve evaluating the association between invasive mechanical ventilation (IMV) status at 72 h and post‐transplant survival (*p* < 0.0001).

IMV status at 72 h was associated with decreased post‐transplant survival. Use of IMV at 72 h was associated with 55% (95% CI 26%–92%) increased hazards of death in adjusted models. Results were similar using restricted mean survival time; subjects requiring IMV at 72 h lived 0.8 months less in the first post‐transplant year (95%CI −1.1 to −0.5 months) and 3.4 months less in the first 3 years (95% CI −4.7 to −2.1) compared to those who were not utilizing IMV at 72 h after adjustment for confounders (Table 2). The largest hazards of IMV‐associated death were seen early post‐transplant. The hazards decreased over time from transplantation as demonstrated by the converging of survival curves over time (Figure 1). In analyses including an interaction term between IMV and time, IMV at 72 h was associated with 2.95‐times the hazards of death at baseline (95% CI 2.10–4.96). The hazards of death decreased by 28% (95% CI 17%–37%) per year post‐transplant relative to those who did not require IMV at 72 h. The association between IMV and survival was modified by severe PGD (*p*‐for interaction 0.002) but not by pre‐transplant ECMO (*p*‐for interaction 0.88) pre‐transplant IMV (*p*‐for interaction 0.92), or transplant type (*p*‐for interaction 0.47). IMV was associated with increased risk of death among those with PGD (HR 2.35, 95% CI 1.43–3.85) but not among those without PGD (HR 1.04, 95% CI 0.77‐1.41). Subjects who required IMV at 72 h but who did not have PGD seemed more likely to have cystic fibrosis but otherwise had similar characteristics to those who required IMV at 72 h but did not have PGD (Table S1).

**TABLE 2:** Association between use of invasive mechanical ventilation (IMV) at 72 h and restricted mean survival time after transplant.

	IMV at 72 h (*n* = 509)	No IMV at 72 h (*n* = 1002)
**Restricted mean survival time (1‐year), months**	10.8 (10.5 to 11.1)	11.6 (11.5 to 11.7)
**Differences in 1‐year restricted life expectancy, months**		
Unadjusted	−0.8 (−1.1 to −0.5)	Ref
Adjusted	−0.8 (−1.1 to −0.5)	Ref
**Restricted mean survival time (3‐years), months**	29.2 (28.1 to 30.2)	32.6 (32.1 to 33.1)
**Differences in 3‐year restricted life expectancy, months**		
Unadjusted	−3.5 (−4.6 to −2.3)	Ref
Adjusted	−3.4 (−4.7 to −2.1)	Ref

*Note:* Adjusted models include covariates for age, pre‐operative ECMO, intra‐operative ECMO, diagnosis group, lung allocation score, intra‐operative transfusion of >1 L packed red blood cells, and center.

### 3.3 IMV and PGD

Both positive and negative predictive value are influenced by the prevalence of the outcome thus we highlight that the incidence of severe PGD at 48 or 72 h of 25% in this cohort. IMV at 72 h had a positive predictive value of 55% for severe PGD at 48/72 h and 40% for severe PGD at 72 h alone. IMV at 72 h had a negative predictive value of 89% for severe PGD at 48/72 h and 94% for severe PGD at 72 h alone.

## 4 Discussion

Requirement of IMV at 72 h is an important early post‐transplant outcome and may be used to prognosticate post‐transplant survival. IMV at 72 h may reflect greater pre‐transplant disease severity as subjects requiring IMV at 72 h had higher lung allocation score at transplantation and were more likely to have required ECMO or mechanical ventilation as a bridge to transplant.

The association between prolonged IMV and survival is consistent with a prior single center study. This association may reflect greater pre‐transplant disease severity. This is consistent with the higher lung allocation score and increased ECMO and mechanical ventilation as a bridge to transplant in those requiring prolonged IMV in our study. This could also reflect greater frailty, a known risk factor for death after transplant. Frail recipients may have greater difficulty recovering from the surgical insult or any post‐operative complications. Prior work has identified that low muscle mass, airway complications, stroke or intracranial bleed, diaphragmatic dysfunction, and pulmonary hypertension are associated with prolonged IMV post‐transplant [2, 16, 17]. Yet it is important to highlight that prolonged IMV did not appear to be entirely attributable to severe PGD. Only 40%–55% of recipients who required IMV at 72 h had severe PGD. This suggests that prolonged IMV may be driven by other indications. Further work is required to understand non‐PGD indications for prolonged IMV.

The association between IMV and survival varied by the presence of severe PGD. Prolonged IMV was not associated with significantly increased risk of death among recipients without PGD. This suggests that severe ischemia reperfusion injury is likely the driver of worse survival. Whether other indications for prolonged IMV might also identify subgroups with worse survival is unknown. Further research investigating this possibility is warranted.

There are limitations to this study. Indications for prolonged IMV are not available. Presence of a pre‐transplant tracheostomy was not recorded; though reassuringly the association between IMV and graft survival was not modified by pre‐transplant IMV suggesting it would be less likely attributable to differences in tracheostomy. There were no standard protocols for assessing extubation readiness across centers. Notably we did account for center in our analyses so any standard protocol within centers should be accounted for in the analysis. We used a 72‐h endpoint based on prior work establishing a median duration of IMV of 48–72 h and our aim was to investigate a pragmatic early endpoint for clinical trials. Whether later timepoints would have similar associations with mortality and PGD, is unknown.

This is consistent with known associations between greater frailty, greater disease severity, and decreased survival after lung transplantation [18, 19]. Importantly, IMV status is a broad indicator of post‐transplant survival and may partially reflect severe PGD, but is not exclusively a measure of lung injury. The association between IMV and survival varies by PGD status with a strong association among those with PGD but not among those without PGD. Further work is required to understand whether other pre‐transplant, peri‐operative, and post‐transplant events uniformly increase duration of IMV post‐transplant or whether the association varies by indication for prolonged IMV.

## Funding

This study was supported by National Institute of Health/NHLBI, Grants: NIH NHLBI K23 HL150280, R01 HL087115, R01 HL155821, K24 HL115354, U01 HL145435, T32 HL007891, K24HL174231.

## Conflicts of Interest

Meghan Aversa reports no conflicts of interest. Shaf Keshavjee reports no conflicts of interest. Tereza Martinu reports no conflicts of interest. Marcelo Cypel reports no conflicts of interest. Andrew T. Sage reports no conflicts of interest. Joshua M. Diamond is a full‐time employee of Glaxo‐Smith Kline. Jonathan P. Singer reports no conflicts of interest. Scott M. Palmer reports research funding to the university from Incyte, AstraZeneca, Bristol‐Myers Squibb, CareDx, and Boerhinger Ingelheim, royalities and licenses from UpToDate, payments for lectures from Altavant Sciences, Bristol Myers Squibb, Boehringer Ingelheim, Mallincrockdt Pharmaceuticals, Abbvie, and Sanofi. Krishna Pandya reports no conflicts of interest. Edward Cantu reports research funding from NIH, XVIVO Inc, CareDx, Pulmocide, Atricure, consulting fees from Lung Bioengineering and United Therapeutics, payment for lecture at the Second China International Lung Transplantation Conference, support for investigator meeting attendance from Pulmocide and CareDx, two patents (US16/308,384, 2017, WO2022087049A1, 2022), leadership roles in the International Society of Heart and Lung Transplantation, UNOS Lung Committee, and the FDA. Jason D. Christie reports grant funding from the National Institutes of Health and the Cystic Fibrosis Foundation, support for attending board meetings from the International Society of Heart and Lung Transplantation, consulting fees from GSK, and participation on the Data Safety Monitoring board for NIH PETALnet and NHLBI. Michaela R. Anderson reports grant funding from NHLBI and payments made to the institution for enrollment in clinical trials for United Therapeutics.

## Supporting information




**Supplemental Table**: Baseline characteristics in subgroups defined by both IMV and severe PGD.
**Supplemental Figure**: Directed acyclic graph for selection of minimal set of covariates demonstrating (A) unadjusted associations and (B) closing of all backdoor paths between exposure and outcome by adjusting for age, ECMO bridge to transplant intra‐op ECMO, diagnosis, intra‐operative blood products, and recipient body mass index.

## Data Availability

The data that support the findings of this study are available on request from the corresponding author. The data are not publicly available due to privacy or ethical restrictions.
