# Supplementary material for: Prolonged Invasive Mechanical Ventilation is Associated With Decreased Survival After Lung Transplantation Among Recipients With Primary Graft Dysfunction: A Lung Transplant Outcomes Group Study
Source: Clin Transplant. 2026 Jan 14;40(1):e70447. doi: 10.1111/ctr.70447 (PMC12801094; doi:10.1111/ctr.70447)

**Prolonged invasive mechanical ventilation is associated with decreased survival after lung transplantation: A Lung Transplant Outcomes Group study**

Meghan Aversa MD MS, Shaf Keshavjee MD MSc, Tereza Martinu MD, Andrew Sage PhD, Joshua Diamond MD MS, Jonathan P Singer MD MS, Scott Palmer MD, Krishna Pandya MD, Ed Cantu MD MS, Jason D Christie MD MS, Michaela R Anderson MD MS

**Supplemental Table:** Baseline characteristics in subgroups defined by both IMV and severe PGD.

**Supplemental Figure:** Directed acyclic graph for selection of minimal set of covariates

**Supplemental Table:** Baseline characteristics in subgroups defined by both IMV and severe PGD.

|  | **IMV at 72 hours** | | **No IMV at72 hours** | |
| --- | --- | --- | --- | --- |
|  | **PGD at 48/72 hours**  **(N=268)** | **No PGD at 48/72 hours**  **(N=221)** | **PGD at 48/72 hours**  **(N=112)** | **No PGD at 48/72 hours**  **(N=877)** |
| Age, years | 57 (47-64) | 60 (47-66) | 60 (48-65) | 61 (52-66) |
| Male sex | 145 (54) | 135 (61) | 64 (57) | 524 (60) |
| LAS at transplant | 47.4 (38.5-67.8) | 47.9 (38.5-80.8) | 44.1 (37.4-57.6) | 39.5 (34.4-47.2) |
| Diagnosis |  |  |  |  |
| Obstructive | 44 (16) | 40 (18) | 16 (14) | 283 (32) |
| Pulm Vascular Disease | 31 (12) | 7 (3) | 5 (4) | 21 (2) |
| Cystic Fibrosis | 20 (7) | 29 (13) | 16 (14) | 125 (14) |
| Restrictive | 172 (64) | 144 (65) | 75 (67) | 447 (51) |
| Height, cm | 170 (160-178) | 170 (163-178) | 170 (161-175) | 170 (163-178) |
| Weight, kg | 76.2 (62.7-86.6) | 72.1 (63.0-85.7) | 76.5 (63.3-88.2) | 72.6 (60.2-83.0) |
| BMI, kg/m^2^ | 26.7 (22.3-29.6) | 25.4 (22.0-29.4) | 26.1 (22.4-29.6) | 24.9 (21.5-28.0) |
| ECMO BTT | 38 (14) | 40 (18) | 10 (0) | 38 (4) |
| Mechanical Ventilation BTT | 34 (13) | 36 (16) | 7 (6) | 34 (4) |
| **Operative Characteristics** |  |  |  |  |
| Bilateral Transplant | 223 (83) | 193 (87) | 89 (79) | 614 (70) |
| Total Ischemic Time, minutes | 626 (468-774) | 667 (525-775) | 555 (369-643) | 526 (331-660) |
| Intra-Op Extracorporeal support | 126 (47) | 105 (48) | 21 (19) | 155 (18) |
| Transfusion > 1 L PRBCs | 166 (62) | 132 (60) | 65 (58) | 523 (60) |
| **Donor Characteristics** |  |  |  |  |
| Male sex | 158 (60) | 113 (52) | 64 (58) | 552 (64) |
| Donor Smoker | 114 (48) | 96 (49) | 49 (46) | 336 (44) |
| Cause of death |  |  |  |  |
| Anoxia | 66 (25) | 61 (28) | 29 (26) | 265 (30) |
| CVA/Stroke | 80 (30) | 77 (35) | 34 (31) | 241 (27) |
| Head Trauma | 105 (39) | 75 (34) | 42 (38) | 327 (37) |
| CNS Tumor | 1 (1) | 1 (1) | 0 (0) | 6 (1) |
| Unknown/Other | 16 (6) | 7 (3) | 7 (6) | 38 (4) |

**Missing**: lung allocation score (3), BMI (2), height (2), weight (2), sex (1), diagnosis group (3), donor sex (3), donor smoking (165)

***Definition of Abbreviations:*** LAS: lung allocation score; IMV: invasive mechanical ventilation; ECMO: extracorporeal membrane oxygenation; BTT: bridge to transplant; PRBCs: packed red blood cells.

**Supplemental Figure:** Directed acyclic graph for selection of minimal set of covariates demonstrating (A) unadjusted associations and (B) closing of all backdoor paths between exposure and outcome by adjusting for age, ECMO bridge to transplant intra-op ECMO, diagnosis, intra-operative blood products, and recipient body mass index.

(A)


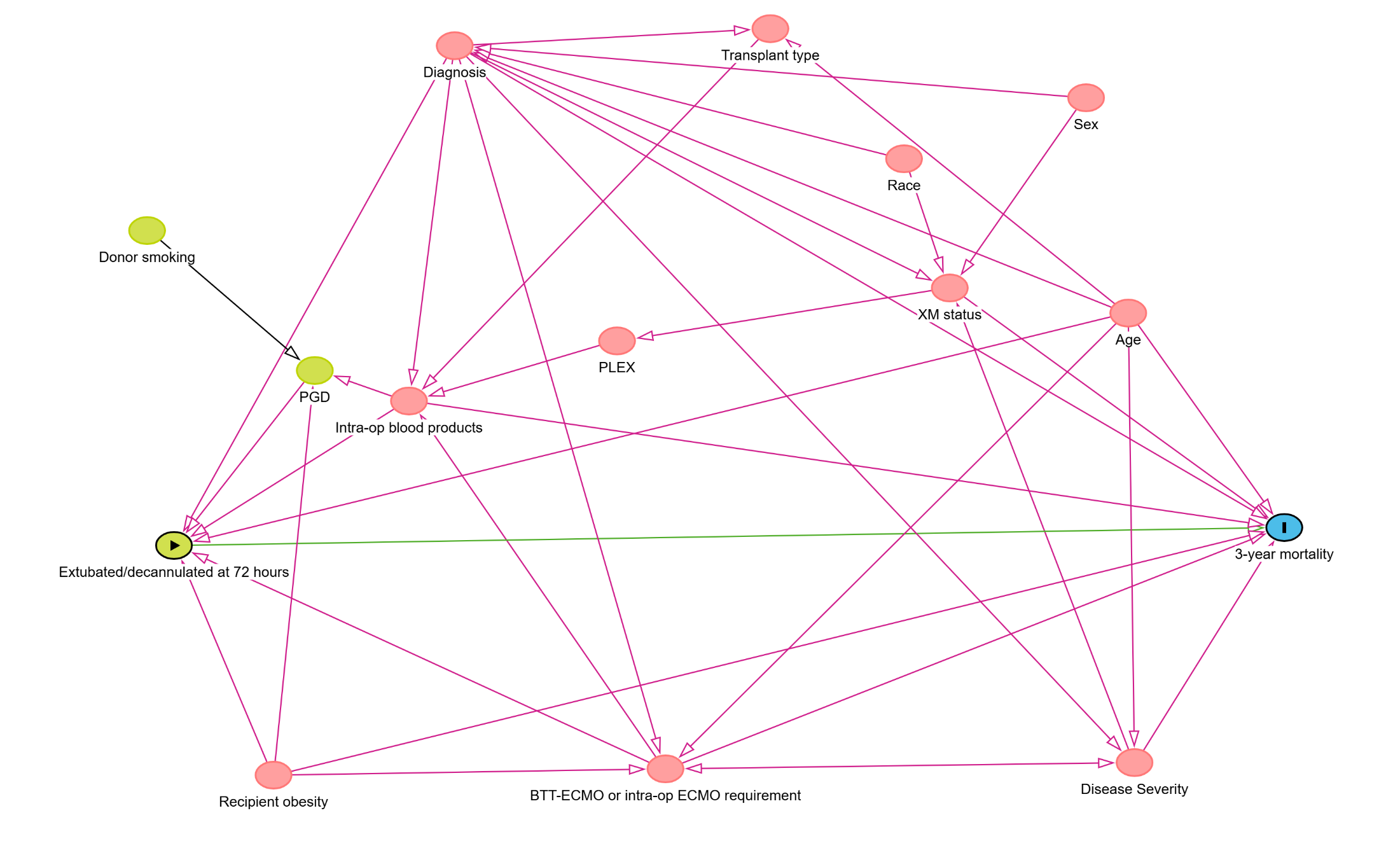


(B)
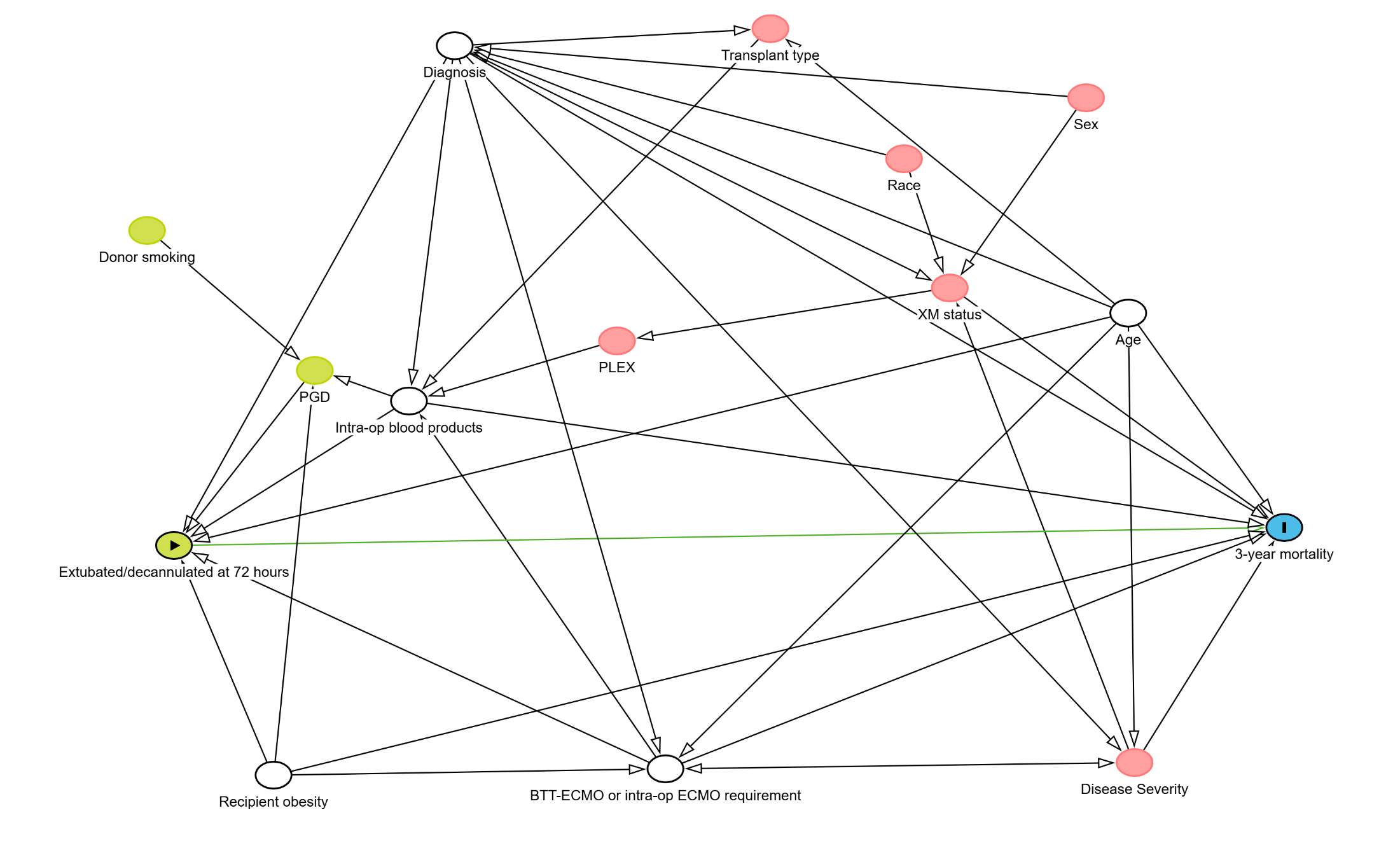

Supplement: Supplementary file 1 — Supplemental Table: Baseline characteristics in subgroups defined by both IMV and severe PGD. Supplemental Figure: Directed acyclic graph for selection of minimal set of covariates demonstrating (A) unadjusted associations and (B) closing of all backdoor paths between exposure and outcome by adjusting for age, ECMO bridge to transplant intra‐op ECMO, diagnosis, intra‐operative blood products, and recipient body mass index. [file CTR-40-e70447-s001.docx]
